# Anti-NAFLD Effect of Djulis Hull and Its Major Compound, Rutin, in Mice with High-Fat Diet (HFD)-Induced Obesity

**DOI:** 10.3390/antiox10111694

**Published:** 2021-10-27

**Authors:** Yu-Tang Tung, Jun-Lan Zeng, Shang-Tse Ho, Jin-Wei Xu, Shiming Li, Jyh-Horng Wu

**Affiliations:** 1 Graduate Institute of Biotechnology, National Chung Hsing University, Taichung 402, Taiwan; peggytung@nchu.edu.tw; 2 Nutrition Research Center, Taipei Medical University Hospital, Taipei 110, Taiwan; 3 Cell Physiology and Molecular Image Research Center, Wan Fang Hospital, Taipei Medical University, Taipei 116, Taiwan; 4 Department of Forestry, National Chung Hsing University, Taichung 402, Taiwan; arashi0221@smail.nchu.edu.tw (J.-L.Z.); ecsgunro@gmail.com (J.-W.X.); 5 Department of Wood Based Materials and Design, National Chiayi University, Chiayi 600, Taiwan; stho@mail.ncyu.edu.tw; 6 Department of Food Science, Rutgers University, New Brunswick, NJ 08901, USA; Shiming@rutgers.edu

**Keywords:** Djulis, nonalcoholic fatty liver disease, rutin, obesity, fatty acid oxidation, anti-inflammation

## Abstract

Nonalcoholic fatty liver disease (NAFLD) has become the main cause of chronic liver disease worldwide, and the increasing trend of NAFLD has burdened the healthcare system. NAFLD encompasses a wide range of liver pathologies, from simple benign hepatocyte steatosis to more severe inflammatory nonalcoholic steatohepatitis. Djulis (*Chenopodium formosanum* Koidz.) is traditionally used as a native cereal and a food supplement that promotes human health through its antioxidant, hepatoprotection, skin protection, hypolipidemic, hypoglycemic, and antitumor effects. Djulis hull, regarded as agricultural waste, is usually removed during food processing and contains high rutin content. The present study evaluated the anti-NAFLD effect of Djulis hull and its major compound, rutin, in mice with high-fat diet (HFD)-induced obesity. Male C57BL/6J mice were randomly divided into one of five diet groups (n = 6 per group) and fed the following for 16 weeks: (1) normal diet group (ND), (2) HFD group (HFD), (3) HFD and oral gavage of low dose (50 mg/kg) of Djulis hull crude extract group (HFD/LCE), (4) HFD and oral gavage of high dose (250 mg/kg) of Djulis hull crude extract group (HFD/HCE), or (5) HFD and oral gavage (50 mg/kg) of rutin (HFD/R) group. We found that Djulis hull crude extract markedly reduced HFD-induced elevation in body weight and fat around the kidney weights, hepatic injury indicators (AST and ALT), and steatosis and hypertrophy. Furthermore, Djulis hull crude extract administration significantly affected DG(20:4/18:1), PA(22:0/17:1), PC(10:0/17:0), and PA(18:4/20:5) in HFD-induced obese mice. In addition, treating HFD-induced obese rats with Djulis hull crude extract significantly increased fatty acid oxidation by increasing the protein expression of phosphorylated AMP-activated protein kinase, peroxisome proliferator-activated receptor-α, and hepatic carnitine palmitoyltransferase-1 in the liver. Moreover, the administration of Djulis hull crude extract significantly decreased the inflammatory response (PPARγ, IL-6, and TNF-α) to modulate oxidative damage. Therefore, Djulis hull crude extract attenuated the progression of NAFLD by reducing inflammation mediated by PPARγ and enhancing the expression levels of genes involved in fatty acid oxidation mediated by AMPK signaling.

## 1. Introduction

Nonalcoholic fatty liver disease (NAFLD) has become the leading cause of liver dysfunction worldwide [1,2]. Patients with NAFLD highly overlap with the development of metabolic syndrome, type 2 diabetes mellitus, and hypertension [2]. NAFLD encompasses a broad range of pathological diseases, including fatty liver, hepatosteatosis, fibrosis, and cirrhosis, which eventually lead to life-threatening hepatocellular carcinoma [3]. However, due to their severe side effects, most approved and marketed anti-obesity drugs have been recalled [4]. Therefore, natural products can be an effective strategy to prevent the onset of NAFLD.

NAFLD most commonly refers to fatty liver resulting from the deposition of excessive lipids characteristic of a hypercaloric diet [3]. High-fat diet (HFD)-induced liver steatosis is mainly caused by increased lipogenesis and decreased fatty acid oxidation [5]. In HFD-induced obese mice, lipogenic transcription factors are upregulated, thereby activating lipogenic target genes and leading to enhanced lipogenesis [6]. In addition, activated acetyl-CoA carboxylase (ACC) inhibits carnitine palmitoyltransferase-1 (CPT1), which catalyzes the rate-limiting step of long-chain fatty acyl-CoA entering the mitochondria, leading to reduced fatty acid oxidation [5,6].

Djulis (*Chenopodium formosanum* Koidz.) is a Chenopodium species native to Taiwan. It is traditionally used as a local cereal and is brewed as a local wine in Taiwan. Djulis contains high levels of starch, dietary fiber, proteins, grain-limited essential amino acids, and phytochemicals (e.g., polyphenols and catechins) [7]. Previous studies have noted that Djulis has antioxidant, hepatoprotective, skin protective, hypolipidemic, hypoglycemic, and antitumor activities [7,8,9,10]. Chen et al. [11] showed that Djulis water extract improves oxidative stress in rats with alcohol-induced liver damage. Chyau et al. [12] showed that Djulis ethanolic extract significantly inhibits lipid accumulation in 3T3-L1 adipocytes by reducing PPARγ, C/EBPα, and SREBP1 gene expression. Chen et al. [10] noted that Djulis ethanolic extract attenuates hyperlipidemia and hyperglycemia in HFD-induced obese mice.

People are accustomed to eating Djulis kernel, therefore, Djulis hull is considered agricultural waste and is usually removed during food processing. However, Djulis hull contains high rutin content [13]. Previous studies have shown that rutin has hepatoprotective [8] and antihypertensive [14] effects and inhibits adipogenesis [12]. Although several studies have been carried out to investigate the biological activity of Djulis, the therapeutic effects of Djulis hull and its major compound, rutin, against HFD-induced NAFLD have not been studied. Therefore, the aim of the present study was to investigate whether oral administration of Djulis hull improved hepatic steatosis in HFD-induced obese mice and to elucidate the potential underlying molecular mechanisms and the effect of Djulis hull on NAFLD in relation to plasma lipidomic profile changes.

## 2. Materials and Methods

### 2.1. Extraction and Identification of the Major Compounds from Djulis

Fresh Djulis kernels and hulls were available in food markets, which were purchased from Sin Fong Farm (Taitung, Taiwan). The dried kernels and hulls were separately soaked in methanol at room temperature for 7 days. The extracts were decanted, filtered under vacuum, concentrated in a rotary evaporator, and then lyophilized. The yields of crude extracts of Djulis kernel and hull were 6.01% and 3.17%, respectively. The hull crude extracts were then extracted successively with *n*-hexane (Hex), ethyl acetate (EtOAc), *n*-butanol (BuOH), and water to yield the Hex (9.2%), EtOAc (10.1%), BuOH (9.1%), and water (52.8%) soluble fractions. Each fraction was tested by various assays to determine the most active fraction. After assays, the phytocompounds of the BuOH soluble fraction from Djulis hull methanolic crude extracts were analyzed using an Orbitrap Fusion Lumos Tribrid mass spectrometer (Thermo Fisher Scientific, San Jose, CA, USA) coupled with an UHPLC (ultra-high-performance liquid chromatography) system (UltiMate 3000 Rapid Separation Dual System, Thermo Fisher Scientific). Briefly, 10 μL of the BuOH soluble fraction of Djulis hull (10 mg/mL in 80% methanol) was subjected to LC-tandem MS analysis. The column oven temperature was 40 °C. The flow rate of the mobile phase (solvent A: H_2_O + 0.1% formic acid, solvent B: acetonitrile + 0.1% formic acid) was 0.4 mL/min. The BuOH soluble fraction of Djulis hull was analyzed with an ACQUITY UPLC BEH C18 column (1.7 µm, 50 × 2.1 mm; Waters, Milford, MA, USA) by the following gradient: 0–1.0 min 5% of B; 1.0–11.0 min 5–100% of B; 11.0–13.0 min 100% of B; 13.0–13.2 min 100–5% of B; 13.2–15.0 min 5% of B. The settings of MS acquisition were as given below, and the ionization was performed by an HESI (heated electrospray ionization) source, with capillary spray voltage at 3.5 kV, vaporizer temperature at 275 °C, ion transfer tube temperature at 300 °C, sheath gas at 40 (arbitrary units, A.U.) and auxiliary gas at 8.0 (A.U.). The data-dependent acquisition method was applied for metabolomic analysis. The scan events contained an MS1 spectrum that was first acquired, followed by eight events of MS2 acquisition to the 8 most intense ions in the MS1 spectrum for each scan segment. The data were acquired in positive ion detection mode at *m*/*z* 100–1500. The spectral resolution was set at 30,000 for MS1 and 15,000 for MS2 acquisition, and the minimal ion intensity was 10^6^ (S/N ratio > 5). The higher-energy collision-induced dissociation (HCD, collision energy = 30 A.U.) method was used in this study to facilitate ion activation. For the molecular networking analysis, the LC-tandem MS data were first converted into mzXML format by MSconvert (http://proteowizard.sourceforge.net, accessed on 22 June 2021) and then processed with mzXML data on Global Natural Products Social Molecular Networking (GNPS, https://gnps.ucsd.edu/ProteoSAFe/static/gnps-splash.jsp, accessed on 22 June 2021). A molecular network was created using the online workflow (https://ccms-ucsd.github.io/GNPSDocumentation/, accessed on 22 June 2021) on the GNPS website (http://gnps.ucsd.edu). The data process of molecular networking was conducted according to previous report [15]. Cytoscape 3.8.2 was used to visualize molecular networks. The molecular networks of the BuOH soluble fraction of Djulis hull extracts are publicly available at https://gnps.ucsd.edu/ProteoSAFe/status.jsp?task=192751dbccb74afaa89725d77e31bd64 (accessed on 22 June 2021). All LC-MS/MS data of the BuOH soluble fraction of Djulis hull extracts (MSV000087873) have been deposited on MassIVE (https://massive.ucsd.edu, accessed on 23 July 2021).

### 2.2. DPPH (1,1-Diphenyl-2-picrylhydrazyl) Assay

The DPPH free radical scavenging activity of Djulis kernel and hull crude extracts and various soluble fractions of Djulis hull were examined according to the method reported by Tung et al. [16]. (+)-Catechin was used as a positive control. The inhibition ratio (percent) was calculated according to the following equation: % inhibition = [(absorbance of control − absorbance of sample)/absorbance of control] × 100.

### 2.3. Determination of Total Phenolics and Total Flavonoids

Total phenolic contents (TPCs) were determined according to the Folin–Ciocalteu method [16] using gallic acid as a standard. The TPCs were expressed as gallic acid equivalents (GAEs) in milligrams per gram sample. Total flavonoid contents (TFCs) were determined by the AlCl_3_ method [17] using rutin as a standard. The TFCs were expressed as quercetin equivalents (QEs) in milligrams per gram sample. Three replicates were made for each test sample.

### 2.4. Animals

Seven-week-old male C57BL/6J mice from the National Laboratory Animal Center (Taipei, Taiwan) were housed in a temperature-controlled (22 ± 2 °C) animal center with a 12:12 h light–dark cycle (light on at 07:00). C57BL/6J related mouse strains are widely used in diet-induced obesity animal models [18]. All animal experiments were approved by the Institutional Animal Care and Use Committee of Taipei Medical University (LAC-2020-0229) and performed in accordance with the NIH guide for the care and use of laboratory animals. The normal diet consisted of 13% fat with an energy density of 2.89 kcal/g (Laboratory Rodent Diet 5001, LabDiet, St. Louis, MO, USA). The HFD contained 21% fat and 0.15% cholesterol with an energy density of 4.67 kcal/g (Diet #D12079B, Research Diets, New Brunswick, NJ, USA). After 1 week of acclimatization, all 30 mice were randomly divided into five treatment groups (n = 6) based on diet: (1) normal diet group (ND), (2) HFD group (HFD), (3) HFD and oral gavage of low dose (50 mg/kg) of Djulis hull crude extract group (HFD/LCE), (4) HFD and oral gavage of high dose (250 mg/kg) of Djulis hull crude extract group (HFD/HCE), and (5) HFD and oral gavage of (50 mg/kg) rutin (HFD/R). Change in body weight and diet consumption were measured every week. The mice were sacrificed after 16 weeks of intervention. At the end of the experiment, all mice were fasted for 12 h and anaesthetized, and blood samples were collected through cardiac puncture. Plasma and serum were obtained by centrifugation at 1500× *g* and 4 °C for 10 min. The liver, epididymal white adipose tissue (eWAT), and perirenal white adipose (pWAT) tissue were collected and weighed. Additionally, pathological histology of liver tissues was fixed in 4% formaldehyde for histopathology examination. All liver was snap-frozen and stored at −80 °C until further analysis.

### 2.5. Blood Biochemical Measurement

Plasma triglyceride (TG), total cholesterol (TC), low-density lipoprotein cholesterol (LDL-C), high-density lipoprotein cholesterol (HDL-C), aspartate aminotransferase (AST), and alanine aminotransferase (ALT) levels were measured using a Roche Modular P800 (Roche Diagnostics, Indianapolis, IN, USA).

### 2.6. Plasma Lipidomic Profile Analysis

Plasma lipids were extracted using the Folch method as previously described [18] with slight modifications. Briefly, 1.5 mL of methanol was added to a 40 µL plasma sample, followed by the addition of 3 mL of CHCl_3_ and incubation for 1 h at room temperature with vortex mixing. Then, 1.25 mL of distilled water was added, and the sample was kept standing for 10 min to facilitate phase separation. The sample was centrifuged for 10 min at 1000× *g* at 4 °C, and 2 mL aliquots of the organic phase were collected followed by vacuum drying to constant weight and stored at −80 °C until subsequent analysis. Finally, lipid extracts were reconstituted in isopropanol/acetonitrile/water (2:1:1, 250 μL) for UPLC-QToF/MS analysis.

MS was performed on a SYNAPT G2 QTof (Waters MS Technologies, Wilmslow, UK). Parameters used for the detection of the positive ionization mode were previously described [19]. In short, the parameters used were as follows: desolvation gas, 900 L/h; desolvation temperature, 550 °C; cone gas, 15 L/h; source temperature, 120 °C; capillary voltage, 2.8 kV; cone voltage, 40 V; and TOF-MS scan range, 100–2000 *m*/*z*. The data acquisition rate was set at 1.2 s with a 0.02 s interscan delay in the Waters MS acquisition mode with full exact masses simultaneously saved by rapidly alternating between two functions. Function 1 acquired data with a low collision energy of 4 and 2 eV for the trap and transfer collision cells, respectively, and function 2 acquired data through a transfer collision energy ramp of 15–35 eV. All scans were acquired by LockSpray to maintain accuracy and reproducibility. We used 1 ng/μL leucine–enkephalin as the LockMass, with a flow rate of 5 μL/min. The LockSpray frequency was set at 20 s, and data were collected in continuum mode. Waters MassLynx v4.1 software (Waters MS Technologies, Manchester, UK) was used to monitor the acquisition of data.

MS data were then processed by Progenesis QI software by using high-resolution positive-ion MS for polar lipids. Alignment, peak picking, and identification of lipids were performed. Data sheets from Progenesis QI software were obtained, and the absolute intensities of all identified compounds were recalculated to determine the relative abundances of lipid molecules. Data were logarithmically transformed to obtain a Gaussian normal distribution, and Pareto scaling was used for the final statistical models. Data were then exported into EZinfo 2.0 software for multivariate statistical analysis. Principal component analysis (PCA) and orthogonal projections to latent structures discriminant analysis (OPLS-DA) were used as final statistical models to determine group clusters. Lipid molecules with the highest effect on group clustering were determined as VIP > 1, FC > 1, and *p* < 0.05.

### 2.7. Histopathology Analysis

Paraffin-embedded liver tissues were sectioned into 4 μm thick sections and then stained with hematoxylin and eosin. Sections were examined by a clinical pathologist under a light microscope equipped with a CCD camera (BX-51, Olympus, Tokyo, Japan).

### 2.8. Western Blot Analysis

The protein expression in the liver was determined through Western blotting following a previously described protocol [20]. The membranes were then incubated with primary antibody (PPARγ, IL-6, IL-1β, SIRT1, LXRα, pAMPKα, AMPKα, PPARα, pACC, ACC, FAS, SREBP1c, CPT1B, and β-actin) at room temperature for 2 h. In this study, the primary antibodies were anti-PPARγ (Cat# 2435S, 1:1000, Cell Signaling, Danvers, MA, USA), anti-IL-6 (Cat# 21865-1-AP, 1:1000, Proteintech, Rosemont, IL, USA), anti-IL-1β (Cat# 16806-1-AP, 1:1000, Proteintech), anti-SIRT1 (Cat# 9475S, 1:1000, Cell Signaling), anti-LXRα (Cat# ab176323, 1:1000, Abcam, Cambridge, UK), anti-pAMPKα (Cat# AF3423, 1:1000, Affinity, San Francisco, CA, USA), anti-AMPKα (Cat# AF6423, 1:1000, Affinity), anti-PPARα (Cat# sc-398394, 1:500, Santa Cruz, Santa Cruz, CA, USA), anti-pACC (Cat# D7D11,1:2000, Cell Signaling), anti-ACC (Cat# C83B10, 1:2000, Cell Signaling), anti-FAS (Cat# C20G5, 1:2000, Cell Signaling), anti-SREBP1c (Cat# ab28481, 1:2000, Abcam), anti-CPT1B(Cat# DF3904, 1:500, Affinity), and anti-β-actin (Cat# GTX109639, 1:10000, Genentech, San Francisco, CA, USA). The relative expression of proteins was quantified densitometrically using ImageJ software (Wayne Rasband, Madison, WI, USA), and β-actin was used as the internal control.

### 2.9. Measurement of TNF-α

Serum TNF-α levels were measured using an ELISA MAXTM MOUSE TNF-α ELISA kit (Cat# 430907, Bio Legend, San Diego, CA, USA).

### 2.10. Statistical Analysis

Data are expressed as the mean ± SEM. The statistical analysis was performed using GraphPad Prism 6.0 (GraphPad Software, San Diego, CA, USA). Differences between groups were analyzed using one-way ANOVA followed by a post hoc Duncan’s test. Significance levels were established at *p* < 0.05.

## 3. Results

### 3.1. Antioxidant Activity and Metabolomic Analysis of Djulis Extracts

The present study showed that the crude extracts of Djulis hull exhibited a better DPPH free radical scavenging activity (IC_50_ value: 32.7 µg/mL) and higher TPC (89.6 ± 3.8 mg of GAE/g) than those of Djulis kernel (IC_50_ value: 324.8 µg/mL, TPC: 28.7 ± 0.5 mg of GAE/g). In addition, among all derived soluble fractions from Djulis hull crude extracts, the BuOH fraction exhibited a better DPPH free radical scavenging performance (IC_50_ value: 19.0 µg/mL), higher TPC (188.1 ± 3.3 mg of GAE/g), and higher TFC (34.6 ± 1.1 mg of QE/g) than Hex (IC_50_ value: >100 µg/mL, TPC: 17.3 ± 0.7 mg of GAE/g, TFC: 13.3 ± 0.2 mg of QE/g), EtOAc (IC_50_ value: 39.1 µg/mL, TPC: 289.0 ± 4.7 mg of GAE/g, TFC: 25.9 ± 0.4 mg of QE/g), and water (IC_50_ value: 38.1 µg/mL, TPC: 84.7 ± 0.9 mg of GAE/g, TFC: 3.8 ± 0.0 mg of QE/g) fractions. To further understand the metabolomic features of Djulis hull extracts, we conducted molecular networking analysis through GNPS. The results were further visualized using Cytoscape. Our results revealed that 1133 nodes (a node represents a precursor ion of a metabolite) and more than 50 molecular clusters (the connected nodes that have structural similarity will be grouped into a cluster) were found in the molecular networking analysis. In this study, 94 metabolites were annotated by the GNPS library (https://gnps.ucsd.edu/ProteoSAFe/status.jsp?task=192751dbccb74afaa89725d77e31bd64, accessed on 22 June 2021).

**Figure 1:**
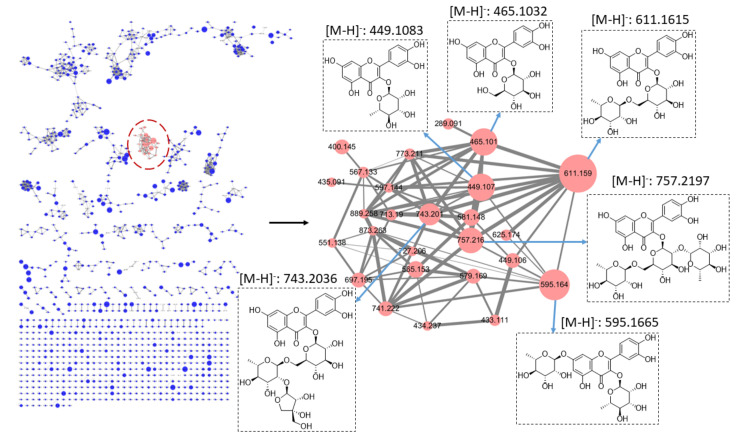
Molecular networking analysis of the BuOH soluble fraction of Djulis hull extract.

Since the antioxidant activity of plant extracts is closely associated with their flavonoid components, we then focused on the screening of flavonoid components in Djulis hull extracts. Interestingly, a cluster comprising 26 nodes related to flavonoid glycosides was observed in the molecular networking (Figure 1). The larger node represents a higher relative abundance of flavonoid glycosides (quercetin derivatives); therefore, six major metabolites (quercetin-3-*O*-rhamnoside, quercetin-3-*O*-glucoside, quercetin-3-*O*-rhamnoside-7-*O*-rhamnoside, quercetin-3-*O*-rutinoside, quercetin-3-*O*-apiosyl-(1→2)-rhamnosyl-(1→6)-glucoside, and quercetin-3-*O*-(2″-rhamnosyl rutinoside)) in the Djulis hull extracts were annotated according to the GNPS library and their MS/MS spectra (Table 1). The largest node, *m*/*z* 611.1615, was identified as quercetin-3-*O*-rutinoside (rutin). Therefore, the anti-NAFLD effects of rutin were further investigated in this study.

### 3.2. Effects of Djulis Hull Crude Extract and Rutin on Body Weight, Liver, eWAT, and pWAT in HFD-Induced Obese Mice

**Figure 2:**
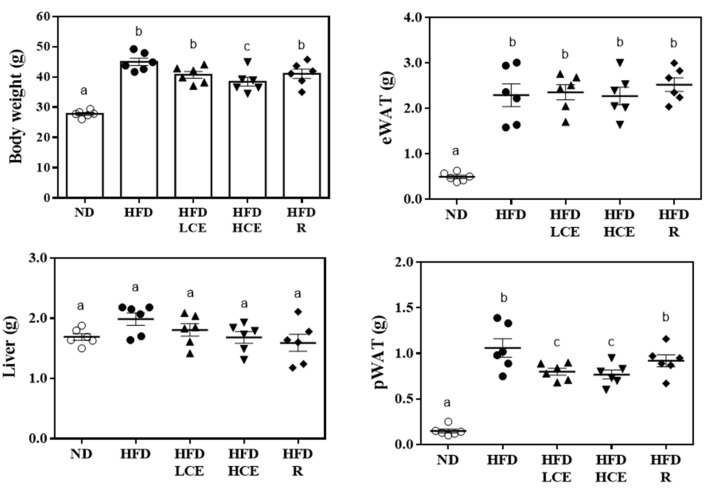
Effect of Djulis hull crude extract and rutin on body weight and tissue weight in HFD-induced obese mice. ND: normal diet; HFD: high-fat diet; LCE: low dosage of crude extract; HCE: high dosage of crude extract; R: rutin; eWAT: epididymal white adipose tissue; pWAT: perirenal white adipose tissue. Values represent the mean ± SEM (n = 6). Statistical methods were used by one-way ANOVA, and the different letters are significantly different at *p* < 0.05.

The average food intake was significantly lower in the HFD-fed groups than in the normal diet-fed group (*p* < 0.05). However, the caloric intake in the HFD-fed groups was significantly higher than that in the normal diet-fed group (*p* < 0.05). There was no significant difference in caloric intake among all HFD-fed groups (HFD, HFD/LCE, HFD/HCE, and HFD/R groups). In addition, the initial body weight of the HFD group did not significantly differ from that of the HFD-fed groups, but the final body weights of the HFD/LCE group were lower than those of the HFD group (*p* < 0.05) (Figure 2). The liver, eWAT, and pWAT were weighed at the end of the study, as shown in Figure 2. The HFD group exhibited significantly increased eWAT and pWAT weights (*p* < 0.05). The pWAT weights in the ND, HFD, HFD/LCE, HFD/HCE, and HFD/R groups were 0.15 ± 0.05, 1.06 ± 0.22, 0.80 ± 0.08, 0.77 ± 0.11, and 0.92 ± 0.15 g, respectively. The pWAT weight was 7.07-fold greater for the HFD group than for the ND group (*p* < 0.05). However, the HFD/LCE and HFD/HCE groups significantly decreased pWAT weights by 24.5% (*p* < 0.05) and 27.4% (*p* < 0.05), respectively, compared to the HFD group.

### 3.3. Effects of Djulis Hull Crude Extract and Rutin on Plasma Biochemical Parameters in HFD-Induced Obese Mice

**Figure 3:**
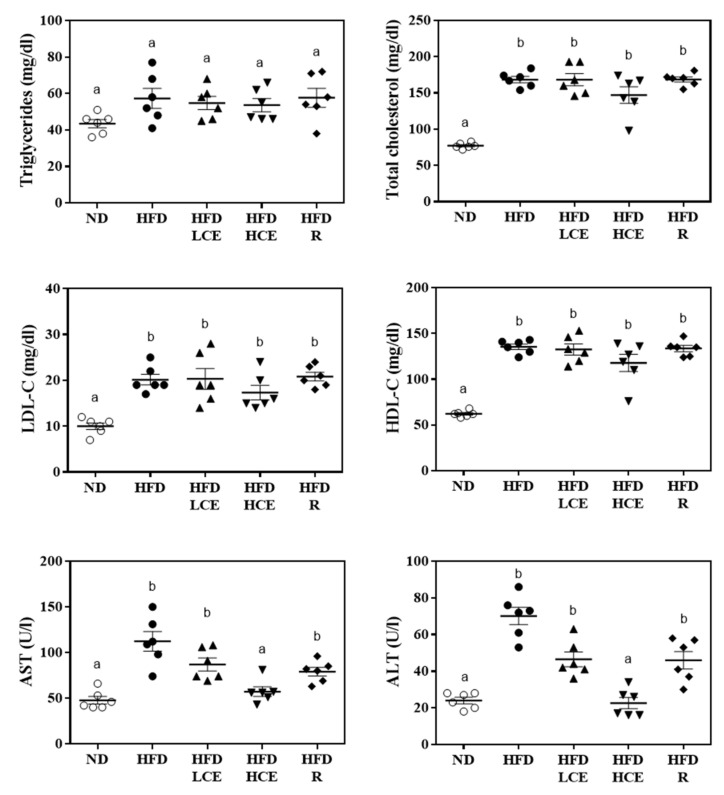
Effect of Djulis hull crude extract and rutin on blood biochemistry in HFD-induced obese mice. ND: normal diet; HFD: high-fat diet; LCE: low dosage of crude extract; HCE: high dosage of crude extract; R: rutin; LDL-C: low-density lipoprotein cholesterol; HDL-C: high-density lipoprotein cholesterol; AST: aspartate aminotransferase; ALT: alanine aminotransferase. Values represent the mean ± SEM (n = 6). Statistical methods were used by one-way ANOVA, and the different superscript letters are significantly different at *p* < 0.05.

Plasma TC, LDL-C, and HDL-C levels in the HFD-fed groups were significantly higher than those in the normal diet-fed group, implying that HFD can increase the serum lipid concentrations, leading to hyperlipidemia (Figure 3). Unexpectedly, Djulis hull crude extract and rutin interventions had no lowering effects on TC, LDL-C, and HDL-C concentrations. Regarding hepatic injury indicators, mice 16 weeks post HFD markedly increased plasma AST and ALT activities in the HFD group compared with the ND group. Moreover, HFD/HCE significantly decreased HFD-induced elevation in plasma AST and ALT activities, indicating that 250 mg/kg Djulis hull crude extract intervention can prevent HFD-induced hepatic injury in mice.

### 3.4. Effects of Djulis Hull Crude Extract on Plasma Lipidomic Profiles in HFD-Induced Obese Mice

**Figure 4:**
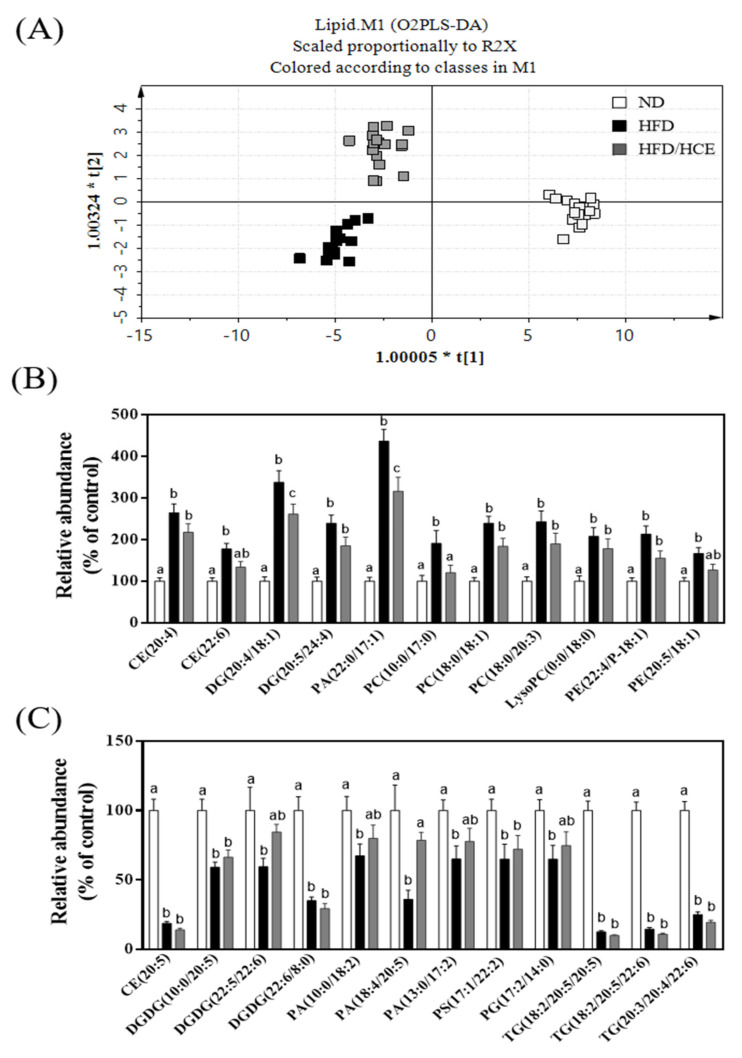
Effect of Djulis hull crude extract on lipidomics in HFD-induced obese mice. (**A**) The orthogonal partial least-squared discriminant analysis (OPLS-DA) score plot for ND, HFD and HFD/HCE groups. The x-and y-axes of OPLS-DA showed the variance explained among the groups. (**B**) Compared with the ND group, the lipid species increased in the HFD group. (**C**) Compared with the ND group, the lipid species decreased in the HFD group. ND: normal diet; HFD: high-fat diet; HCE: high dosage of crude extract. Values represent the mean ± SEM (n = 6). Statistical methods were used by two-way ANOVA, and the different letters are significantly different at *p* < 0.05.

A dramatic change in plasma lipidomic profiles is shown in Figure 4. As shown in Figure 4A, the orthogonal partial least-squared discriminant analysis (OPLS-DA) results indicated that the plasma lipidomic profiles of ND, HFD, and HFD/HCE groups were clustered into three distinct groups, revealing that HFD and Djulis hull crude extract intervention may affect the lipid metabolism in mice. On validation by permutation tests, we obtained OPLS-DA with R2 = 0.939 and Q2 = 0.910, which could be considered good as the standard with R2 = 0.7 and Q2 = 0.4 for biological data. HFD may affect the lipid metabolism in mice. HFD significantly increased 11 species of lipids: CE(20:4), CE(22:6), DG(20:4/18:1), DG(20:5/24:4), PA(22:0/17:1), PC(10:0/17:0), PC(18:0/18:1), PC(18:0/20:3), LysoPC(0:0/18:0), PE(22:4/P-18:1), and PE(22:5/18:1) (Figure 4B). However, significant reductions of 12 lipid species—namely, CE(20:5), DGDG(10:0/20:5), DGDG(22:5/22:6), DGDG(22:6/8:0), PA(10:0/18:2), PA(18:4/20:5), PA(13:0/17:2), PS(17:1/22:2), PG(17:2/14:0), TG(18:2/20:5/20:5), TG(18:2/20:5/22:6), and TG(20:3/20:4/22:6) were identified in HFD-fed mice (Figure 4C). However, Djulis hull crude extract intervention significantly reversed four lipid species affected by HFD: DG(20:4/18:1), PA(22:0/17:1), PC(10:0/17:0), and PA(18:4/20:5).

### 3.5. Effects of Djulis Hull Crude Extract and Rutin on Hepatic Steatosis in HFD-Induced Obese Mice

**Figure 5:**
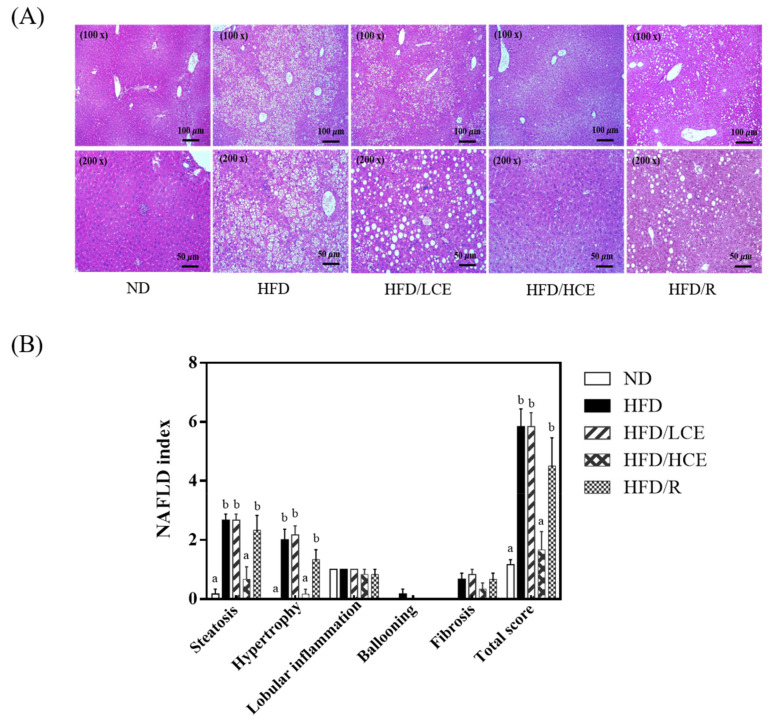
Effect of Djulis hull crude extract and rutin on pathological indices in HFD-induced obese mice. (**A**) Representative hematoxylin and eosin staining of liver sections. (**B**) NAFLD index calculated from individual scores for steatosis, hypertrophy, lobular inflammation, ballooning and fibrosis. ND: normal diet; HFD: high-fat diet; LCE: low dosage of crude extract; HCE: high dosage of crude extract; R: rutin. Values represent the mean ± SEM (n = 6). Statistical methods were used by one-way ANOVA, and the different letters are significantly different at *p* < 0.05.

Histological evidence revealed that the HFD diet-fed mice developed fatty livers with a higher degree of microvesicular fatty changes, steatosis, and hypertrophy than the normal diet-fed mice (Figure 5). The steatosis and hypertrophy scores were dramatically elevated in the HFD group compared with the ND group (*p* < 0.05). Moreover, HFD/HCE significantly decreased HFD-induced elevation in steatosis and hypertrophy (*p* < 0.05), indicating that 250 mg/kg Djulis hull crude extract intervention can prevent HFD-induced hepatic steatosis in mice.

### 3.6. Effects of Djulis Hull Crude Extract on the Anti-Inflammatory Pathway in HFD-Induced Obese Mice

**Figure 6:**
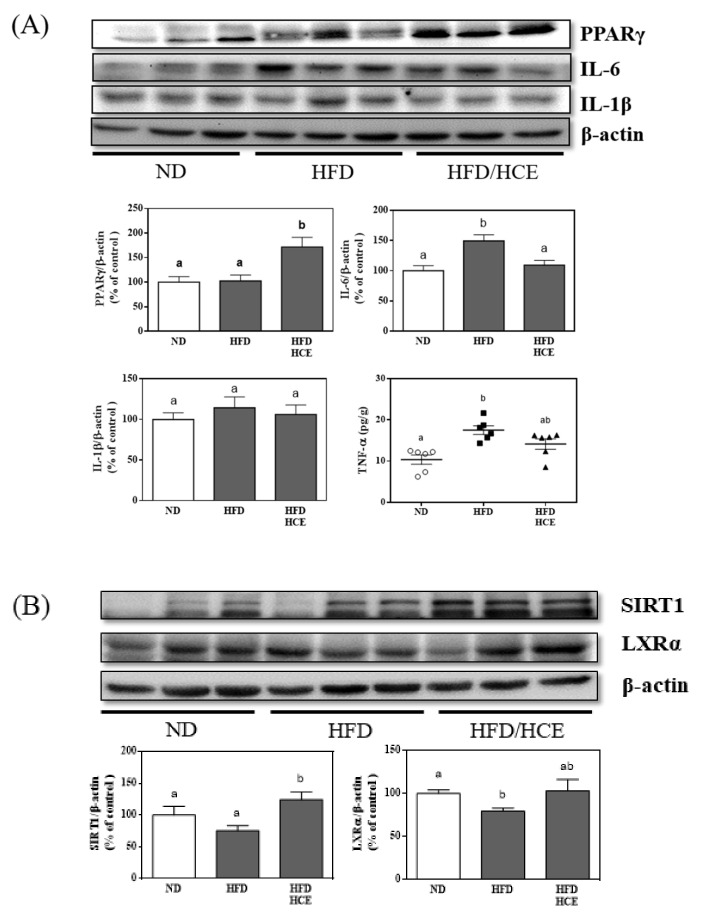

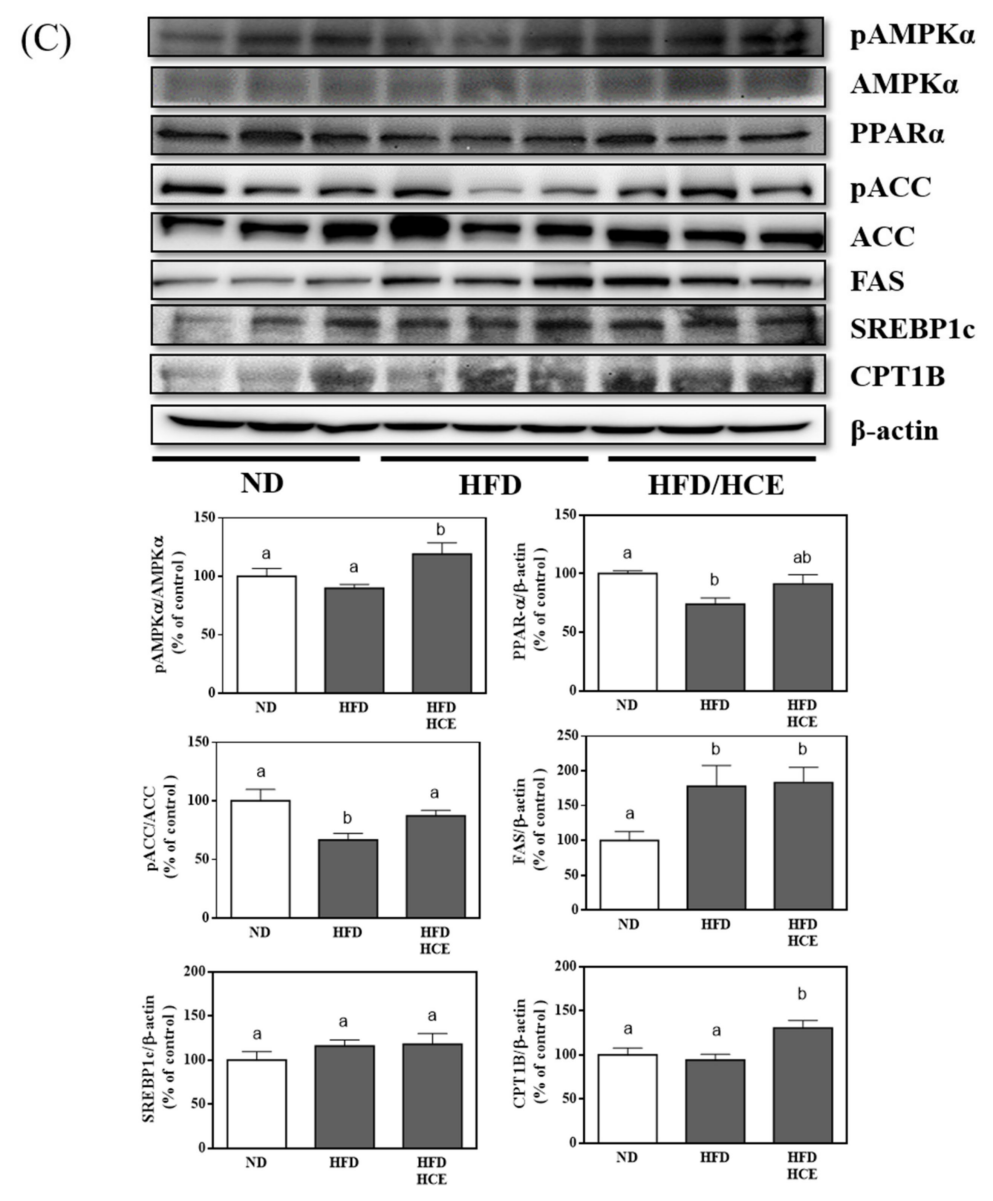
Effect of Djulis hull crude extract on the protein expression of (**A**) PPARγ, IL-1β, IL-6, and TNF-α, (**B**) SIRT1 and LXRα, and (**C**) lipogenesis and β-oxidation pathways in the livers of HFD-induced obese mice. ND: normal diet; HFD: high-fat diet; HCE: high dosage of crude extract. Values represent the mean ± SEM (n = 6). Statistical methods were used by one-way ANOVA, and the different letters are significantly different at *p* < 0.05.

To confirm the mechanism by which Djulis hull crude extract ameliorates hepatic steatosis, the expression of hepatic proteins involved in the anti-inflammatory signaling pathway was evaluated, as shown in Figure 6A. There was no significant difference in IL-1β among the ND, HFD, and HFD/HCE groups. PPARγ protein levels were remarkably increased in the HFD/HCE group compared with the HFD group. The hepatic levels of IL-6 and TNF-α in the HFD group were significantly elevated by 49.4% (*p* < 0.05) and 69.1% (*p* < 0.05), respectively, compared to those in the ND group, indicating that HFD caused a typical liver failure inflammatory response. However, the protein expression levels of IL-6 and TNF-α were significantly decreased in the HFD/HCE group by 26.9% (*p* < 0.05) and 19.2% (*p* < 0.05), respectively, compared to the HFD group. These data indicated that 250 mg/kg Djulis hull crude extract intervention can alleviate HFD-induced hepatic steatosis by upregulating PPARγ, thereby reducing inflammation.

### 3.7. Effects of Djulis Hull Crude Extract on the Relative Protein Expression of SIRT1 and LXRα in HFD-Induced Obese Mice

The protein levels of SIRT1 and LXRα in the HFD group were decreased by 24.6% (*p* < 0.05) and 20.5% (*p* < 0.05), respectively, compared to those in the ND group. However, SIRT1 and LXRα protein expression increased in the HFD/HCE group by 65.2% (*p* < 0.05) and 29.6% (*p* = 0.12), respectively, compared to the HFD group (Figure 6B).

### 3.8. Effects of Crude Djulis Hull Extract on Hepatic Lipogenesis- and Lipolysis-Related Protein Expression in HFD-Induced Obese Mice

To confirm the mechanisms of Djulis hull crude extract amelioration of hepatic steatosis, the expression of hepatic genes involved in lipogenesis and fatty acid β-oxidation was evaluated. pACC was significantly decreased in the hepatocytes of the HFD group (*p* < 0.05), and pAMPK and pACC were markedly elevated in the HFD/HCE group (*p* < 0.05) (Figure 6C). Then, we evaluated the proteins involved in lipogenesis. The protein expression of FAS in the livers of the HFD group was markedly increased compared with that in the livers of the ND group; however, there was no difference between the HFD and HFD/HCE groups. No significant difference in SREBP1c expression was observed among the ND, HFD, and HFD/HCE groups. Moreover, the HFD/HCE group exhibited increases in the protein expression of PPARα and CPT1B, which are involved in fatty acid β-oxidation, compared to the HFD group.

## 4. Discussion

The prevalence of NAFLD is closely related to the rising trend of obesity, which essentially burdens the global health care system. Excessive consumption of high-energy-density foods, such as high-fat or high-sugar diets, increases white adipose tissue, causing metabolic, hormonal, and inflammatory changes, leading to organ damage [21]. Our study revealed that the HFD intervention group had higher body weight, higher white tissue weight, and plasma concentrations of TC, LDL-C, HDL-C, AST, and ALT. Moreover, morphometric analysis revealed liver steatosis and hypertrophy due to the HFD. Djulis hull crude extract and rutin intervention had no notable effect on lowering the blood lipid concentrations. However, Djulis hull crude extract significantly decreased HFD-induced elevation in body weight, pWAT, hepatic injury indicators (AST and ALT), steatosis and hypertrophy. Zhang et al. [22] showed that HFD can alter hepatic function, leading to hepatic injury. In clinical and animal studies, the common biochemical pattern observed in hepatic steatosis caused by NAFLD has elevated blood AST and ALT levels [23]. In our study, an increase in AST and ALT levels accompanied by the histological observation of severe lipid droplets infiltrating the hepatocytes conforms with the findings of previous studies [23]. Our results demonstrated that Djulis hull crude extract significantly reduced plasma AST and ALT levels, indicating that Djulis hull crude extract may ameliorate the negative effects of HFD on the liver and minimize hepatic injury.

Lipidomics is an analytical skill based on liquid mass spectrometry (MS) used to determine the relationship between lipid species and metabolic diseases [7]. Based on the high-resolution output of lipid MS, varying lipid maps derived from the application of different interventions can provide a better understanding of a disease. To understand plasma lipidomic profile changes in different interventions, we analyzed plasma samples by using UPLC-QToF-MS. HFD significantly increased 11 species of lipids: CE(20:4), CE(22:6), DG(20:4/18:1), DG(20:5/24:4), PA(22:0/17:1), PC(10:0/17:0), PC(18:0/18:1), PC(18:0/20:3), LysoPC(0:0/18:0), PE(22:4/P-18:1), and PE(22:5/18:1). However, significant reductions of 12 lipid species—namely, CE(20:5), DGDG(10:0/20:5), DGDG(22:5/22:6), DGDG(22:6/8:0), PA(10:0/18:2), PA(18:4/20:5), PA(13:0/17:2), PS(17:1/22:2), PG(17:2/14:0), TG(18:2/20:5/20:5), TG(18:2/20:5/22:6), and TG(20:3/20:4/22:6)—were identified in HFD-fed mice. A previous study [19] showed that rats fed an HFD for 8 weeks significantly increased eight lipid species: PC(18:2/22:6), DG(18:2/16:0), DG(18:2/18:1), DG(18:1/16:0), CE(20:5), CE(28:2), TG(18:0/16:0/18:3), and glycerol-1-2-hexadecanoate 3-octadecanoate. PC is a hydrophilic lipid required for packaging and export of neutral lipids, such as TGs, in very-low-density lipoprotein (VLDL). Thus, impaired PC biosynthesis reduces VLDL synthesis and secretion, thereby contributing to the development of NAFLD [24]. Increases in the total blood PC in patients with NAFLD were reported [25]. PC was positively correlated with blood and hepatic cholesterol concentrations [19]. However, notably, a study discovered that less unsaturated TG species were located mainly in hepatocyte lipid droplets in mice with liver steatosis [26]. In our study, we determined that plasma PC concentrations were elevated and polyunsaturated TG lipid species were decreased in the NAFLD model group, corresponding with the findings of previous studies [19,25,26]. However, Djulis hull crude extract intervention significantly reversed four lipid species affected by HFD: DG(20:4/18:1), PA(22:0/17:1), PC(10:0/17:0), and PA(18:4/20:5).

NAFLD is always accompanied by inflammation, which plays a vital role in the progression of hepatic steatosis [27]. In our study, we found that Djulis hull crude extract upregulated PPARγ protein levels, which led to a reduction in downstream inflammatory factors involving IL-6 and TNF-α signaling (Figure 6). Previous studies have shown that PPARγ inhibits NFκB-mediated gene activation, thereby reducing the production of proinflammatory mediators [28]. In addition, PPARγ is the main nuclear receptor that regulates lipid metabolism and increases lipid breakdown and mobilization [29]. Mahali et al. [29] and Luo et al. [30] showed that mangiferin and rosiglitazone attenuate hepatic lipid accumulation in HFD-fed mice by activating PPARγ. In contrast, Andrade et al. [31] and Jeon et al. [32] reported that resveratrol and genistein reduce hepatic steatosis in HFD-fed mice by repressing PPARγ. In this study, we found that Djulis hull crude extract could activate PPARγ to reduce inflammatory factors and hepatic lipid accumulation, indicating that Djulis hull crude extract could activate PPARγ to improve hepatic steatosis in HFD-fed mice.

SIRT1 plays an important role in NAFLD [33]. Djulis hull crude extract could increase SIRT1 and LXR. The activation of LXR could inhibit the uptake of cholesterol in the intestines, promote the reverse transport of cholesterol, and reduce inflammation [34]. SIRT1 acts downstream of the AMPK signal and promotes fatty acid catabolism [35].

**Figure 7:**
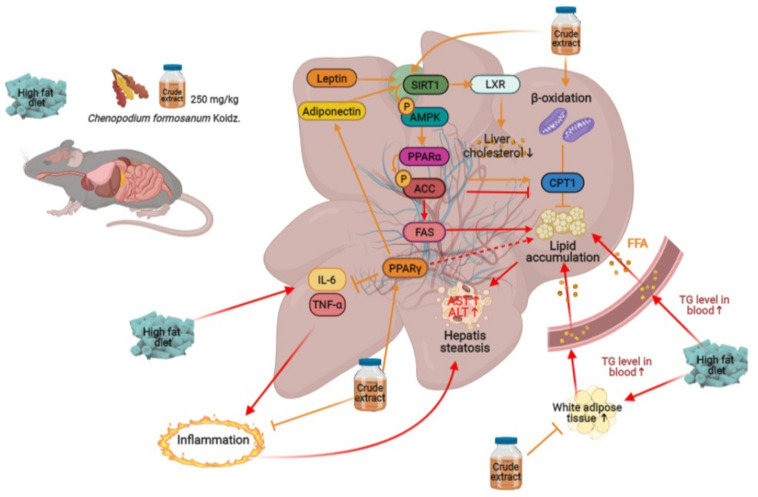
Proposed mechanism of Djulis hull crude extract-mediated modulation of the lipid metabolism pathway in HFD-induced obese mice. The effects of Djulis hull crude extract on the fatty liver of mice may occur through anti-inflammation and the lipid oxidative pathway.

NAFLD is believed to be the result of two major metabolic disorders: increased liver fat production and decreased fat consumption [36]. AMPK is the main regulator of cellular energy homeostasis, which regulates the lipid and glucose metabolism of hepatocytes [37]. Therefore, AMPK is a therapeutic target for the treatment of hepatic steatosis, which could prevent hepatic steatosis by repressing fatty acid synthesis and enhancing fatty acid oxidation [38]. In our study, we found that Djulis hull crude extract could increase pAMPK, PPARα, p-ACC, and CPT1B. Therefore, Djulis hull crude extract could phosphorylate AMPK and inactivate ACC, thereby reducing the production of malonyl-CoA, a precursor for fatty acid synthesis that is also a valid inhibitor of CPT1B, the rate-limiting enzyme in fatty acid oxidation (Figure 7). PPARα controls fatty acid transport and β-oxidation to reduce lipid storage and reduces inflammatory responses in the liver [39]. In this study, we found that Djulis hull crude extract markedly increased the expression of SIRT1, pAMPK, PPARα, pACC, and CPT1B but not the expression of FAS and SREBP1c in the livers of HFD-induced obese mice.

## 5. Conclusions

The present study demonstrated that HFD intervention had higher body weight, higher white tissue weight, plasma concentrations of TC, LDL-C, HDL-C, AST, and ALT, as well as liver steatosis and hypertrophy. Djulis hull crude extract and rutin intervention had no notable effect on lowering the blood lipid concentrations. However, Djulis hull crude extract significantly decreased HFD-induced elevation in body weight, pWAT, hepatic injury indicators (AST and ALT), steatosis and hypertrophy. The effect of Djulis hull crude extract on hepatic steatosis is likely due to reduced inflammation mediated by PPARγ and enhanced expression levels of proteins involved in fatty acid oxidation mediated by AMPK signaling. Djulis has been used as an edible plant in Taiwan for a long time. Therefore, an effective food-assisted therapeutic approach could be developed to treat NAFLD.

## Figures and Tables

**Table 1:** Six major metabolites in the Djulis hull extracts were annotated according to the GNPS library and their MS/MS spectra.

Compound	R.T. (min)	Exact MS (*m*/*z*)	Observed MS (*m*/*z*)	MS Error (ppm)	MS2 (*m*/*z*)	Tentative Compounds
**1**	4.10	449.1084	449.1083	0.22	303.0494	Quercetin-3-*O*-rhamnoside
**2**	4.11	465.1033	465.1032	0.22	303.0490	Quercetin-3-*O*-glucoside
**3**	4.29	595.1663	595.1665	−0.34	449.1072 303.0493	Quercetin-3-*O*-rhamnoside-7-*O*-rhamnoside
**4**	4.07	611.1612	611.1615	−0.49	465.1024 303.0491	Quercetin-3-*O*-rutinoside (Rutin)
**5**	4.02	743.2035	743.2036	−0.13	465.1026 303.0491 133.0487 85.0277	Quercetin-3-*O*-apiosyl-(1→2)-rhamnosyl-(1→6)-glucoside
**6**	3.74	757.2191	757.2197	−0.79	465.1019 303.0491 129.0541 85.0279	Quercetin-3-*O*-(2″-rhamnosyl rutinoside)

## Data Availability

Data is contained within the article.
